# Lasp1 promotes malignant phenotype of non-small-cell lung cancer via inducing phosphorylation of FAK-AKT pathway

**DOI:** 10.18632/oncotarget.20527

**Published:** 2017-08-24

**Authors:** Xiupeng Zhang, Yang Liu, Chuifeng Fan, Liang Wang, Ailin Li, Haijing Zhou, Lin Cai, Yuan Miao, Qingchang Li, Xueshan Qiu, Enhua Wang

**Affiliations:** ^1^ Department of Pathology, Basic Medicine Science and First Hospital of China Medical University, Shenyang, China; ^2^ Department of Radiotherapy, First Hospital of China Medical University, Shenyang, China

**Keywords:** Lasp1, NSCLC, proliferation and invasion, FAK, AKT

## Abstract

Lasp1 (LIM and SH3 domain protein 1) promotes tumor proliferation and invasion in multiple cancer entities including non-small cell lung cancer (NSCLC). However, the molecular mechanism is uncertain to date. In the present study, using immunohistochemistry, we found that Lasp1 expression was significantly correlated with tumor size (*P*=0.005), advanced TNM stage (*P*=0.042), positive regional lymph node metastasis (*P*=0.034) and poor overall survival (*P*<0.001). Similar results were seen in patients with squamous cell lung carcinoma (*P*=0.003 for larger tumor size, *P*=0.017 for advanced TNM stage, *P*=0.003 for positive lymph node metastasis and *P*<0.001 for poor overall survival) but not in patients with lung adenocarcinoma (*P*>0.05). Proliferation and invasion assay showed that Lasp1 dramatically promoted the ability of proliferation and invasion of NSCLC cells. Subsequent western blot results revealed that Lasp1 promoted the expression of Cyclin A2, CyclinB1, and Snail, and inhibited the expression of E-cadherin. Lasp1 directly interacted with FAK and facilitated the expression of phosphorylated FAK (Tyr397) and AKT (Ser473). Incorporation of both FAK inhibitor and AKT inhibitor counteracted the upregulating expression of Cyclin A2, CyclinB1, and Snail, and downregulating expression of E-cadherin expression induced by Lasp1 overexpression. Interestingly, inhibition of FAK signaling pathway attenuated the phosphorylation of AKT, but inhibition of AKT signaling pathway did not affect the phosphorylation of FAK. In conclusion, Lasp1 facilitated tumor proliferation and invasion of NSCLC through directly binding to FAK and enhancing the phosphorylation of FAK (Tyr397) and AKT (Ser473). Lasp1 may be a novel therapeutic target in the treatment of NSCLC patients.

## INTRODUCTION

Lasp1 (LIM and SH3 domain protein 1), localized on chromosome 17q11-21.3, was first identified in 1995 [1]. It contains an N-terminal LIM domain and a C-terminal SRC homology region 3 (SH3) domain [2–4]. The LIM domain is composed of two sequential zinc-binding modules, which are responsible for binding with DNA [5]. The SH3 domain is a 60 amino acids segment shared by diverse structural and signaling proteins [6]. Previous studies had confirmed that Lasp1 was significantly overexpressed in multiple malignant tumors including non-small-cell lung cancer (NSCLC) and enhanced tumor proliferation, invasion and metastasis [7–13]. Fahrmanna et al. found that Lasp1 may be served as negative survival predictor in lung adenocarcinoma patients [14]. Zheng et al demonstrated that Lasp1 promoted tumor proliferation and aggressiveness in NSCLC and thereby predicted poor prognosis of lung cancer patients [15]. Lasp1 has been shown to be a target of upstream regulators or signaling pathways including TGFβ/Smad and multiple miRNAs [4, 15–20]. Shao et al. showed that Lasp1 interacted with 14-3-3σ and decreased the expression of 14-3-3σ in colorectal cancer, which was a suppressor of PI3K/AKT signaling pathway [21]. Wang et al. reported that Lasp1 could function as a key mediator of EMT via inducing phosphorylation of MAPK, PI3K/AKT and Smad signaling in colorectal carcinoma [22]. However, in NSCLC, the direct evidence that elucidates the downstream signaling pathway of Lasp1 is still uncertain.

In this study, we explored the protein level and subcellular distribution of Lasp1 in both lung cancer tissues and cell lines, as well as their clinicopathological relevances. We also investigated the effects of Lasp1 on tumor proliferation and invasiveness after transfected with Lasp1 plasmid or Lasp1-siRNA. In conclusion, we identified that Lasp1 enhanced proliferation and invasion of NSCLC cells through facilitating the activation of FAK-AKT signaling pathway.

## RESULTS

### Lasp1 was overexpressed in NSCLC samples and cell lines

**Figure 1:**
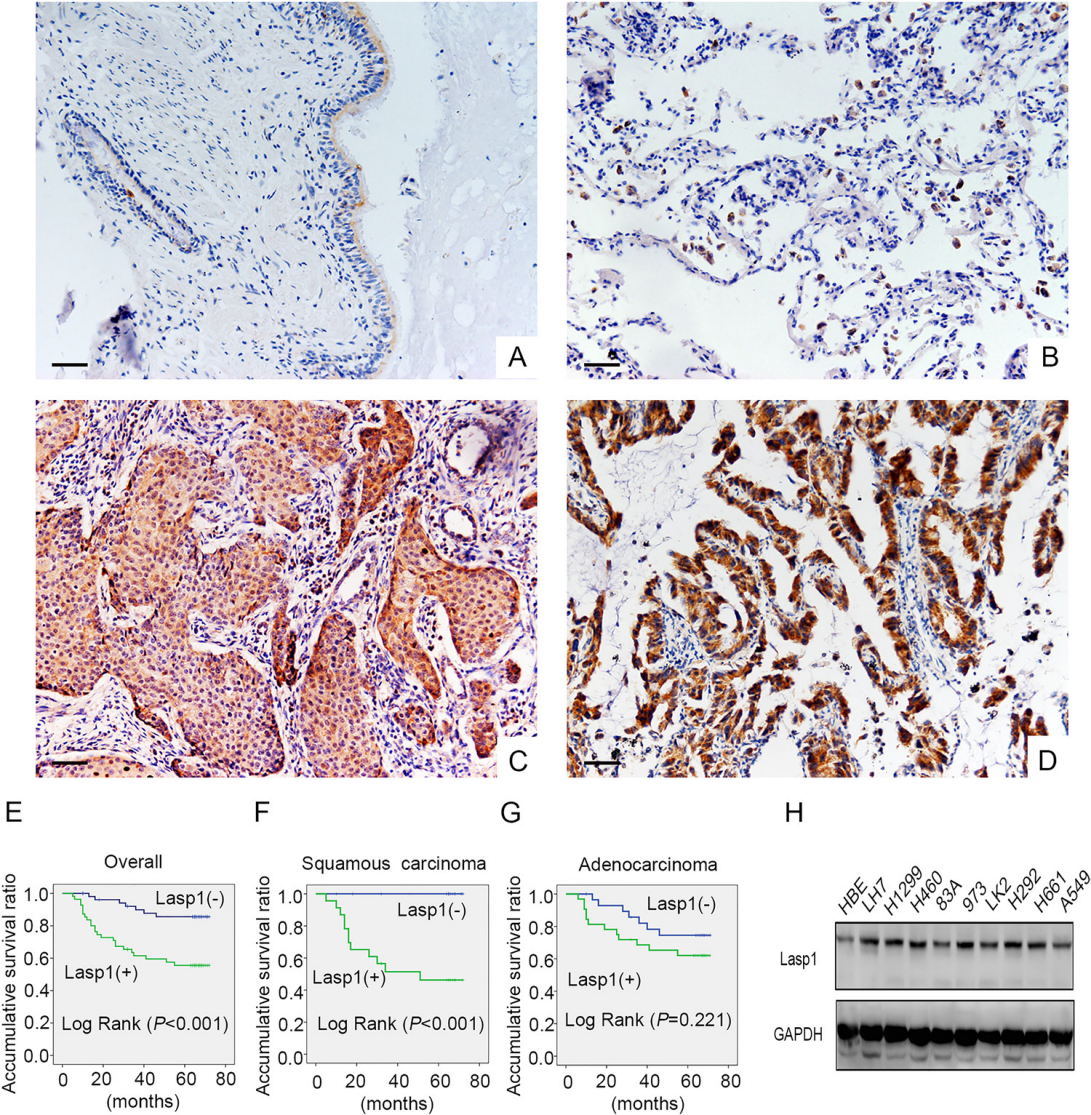
Lasp1 expression in NSCLC specimens and cell lines Lasp1 was negatively expressed in normal bronchial **(A)** and alveolar epithelial **(B)**, however, Lasp1 presented positive expression in the cytoplasm of lung squamous cell carcinoma **(C)** and adenocarcinoma **(D)**. Magnification, 400×; scale bar=50μm. Results of Kaplan–Meier survival analysis in the cohorts of all the NSCLC patients **(E)**, squamous cell lung carcinoma patients **(F)** and lung adenocarcinoma patients **(G)**. In all 9 NSCLC cell lines, the protein level of Lasp1 was higher than that in HBE cells **(H)**.

Initially, immunohistochemisty assay was employed in 109 cases NSCLC and 68 cases corresponding noncancerous lung tissues. We found that Lasp1 presented negative or only dim expression in normal lung samples (Figure 1A and 1B), whereas Lasp1 was highly expressed in the cytoplasm of NSCLC (Figure 1C and 1D). The positive ratio of Lasp1 in noncancerous lung tissues (14.7%, 10/68) was obviously lower than that in NSCLC tissues (50.5%, 51/109, *P*<0.001). Statistical analysis results showed that positive expression of Lasp1 was significantly correlated with larger tumor size (*P*=0.005), advanced TNM stage (*P*=0.042) and positive lymph node metastasis (*P*=0.034). There were no correlations between Lasp1 expression and age, sex, histological type and differentiation (*P*>0.05, Table 1). In addition, we also analyzed the association between Lasp1 and clinicopathological features in lung adenocarcinomas or squamous cell lung carcinomas, separately. Similar results were also seen in squamous cell lung carcinoma (*P*=0.003 for larger tumor size, *P*=0.017 for advanced TNM stage and *P*=0.003 for positive lymph node metastasis; Supplementary Table 1), whereas in lung adenocarcinomas, overexpression of Lasp1 showed no significant associations with clinicopathological features, as well as with different histological subtypes, including TRU (terminal respiratory unit) status, EGFR mutation and KRAS mutation (Supplementary Table 2). Kaplan–Meier analysis results showed that the overall survival time of patients with positive Lasp1 expression (49.453 ± 3.637 months) was significantly shorter than those with negative or weak Lasp1 expression (65.997 ± 2.169 months, *P*<0.001, Figure 1E). Furthermore, in patients with squamous cell lung carcinoma, positive Lasp1 expression also indicated adverse clinical outcome (*P*<0.001, Figure 1F). In lung adenocarcinoma, there was no significant association of the overall survival time of patients with positive Lasp1 expression and negative Lasp1 expression (*P*=0.221, Figure 1G). We also performed western blot to explore Lasp1 expression in NSCLC cell lines. The results indicated that Lasp1 was positively expressed in all 10 cell lines and showed higher protein level in 9 lung cancer cells than in HBE cells (Figure 1H).

**Table 1:** Correlation of the expression of Lasp1 with clinicopathological features in 109 cases of NSCLC

Clinicopathological factors	N	Positive	Negative	χ2	*P*
Age (years)					
<60	60	31	29	0.078	0.848
≥60	49	24	25		
Gender					
Male	68	38	30	2.127	0.169
Female	41	17	24		
Histological type					
Squamous cell carcinoma	47	19	28	0.033	1.000
Adenocarcinoma	62	24	38		
Tumor Size					
>3cm	68	27	41	8.362	**0.005**
≤3cm	41	28	13		
Differentiation					
Well	38	19	19	0.05	1.000
Moderate+Poor	71	36	35		
TNM classification					
I+II	83	37	46	4.813	**0.042**
III	26	18	8		
Lymph node metastasis					
Positive	48	30	18	4.975	**0.034**
Negative	61	25	36		

### Overexpression of Lasp1 enhanced proliferation and invasion of NSCLC cells

**Figure 2:**
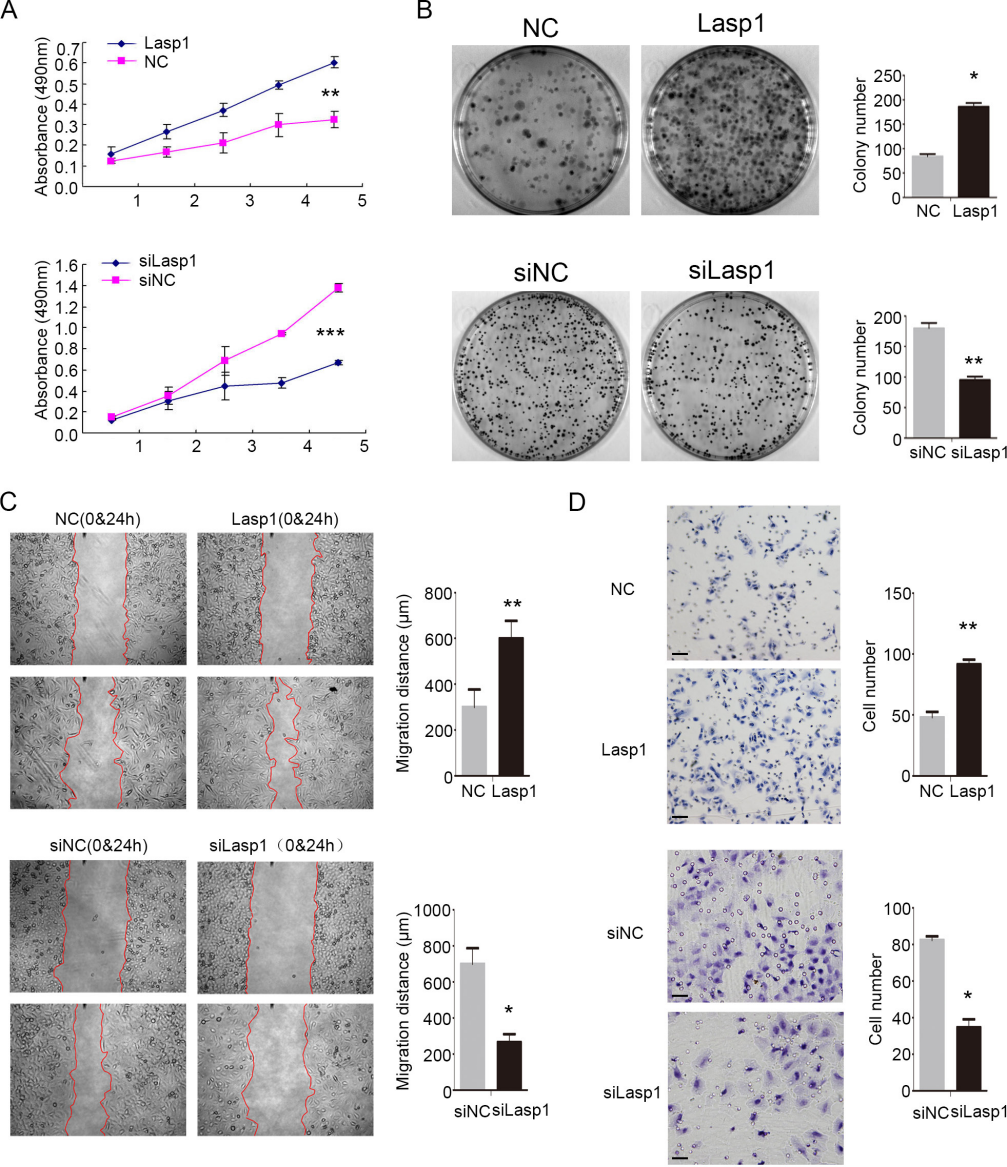
Lasp1 enhanced NSCLC cell growth, invasion and migration As measured by MTT assay, the proliferation ratio was increased after Lasp1 overexpression in A549 cells, and reduced after the Lasp1 knockdown in H460 cells **(A)**. Colony formation assay showed that Lasp1 overexpression significantly increased the number and the size of foci in A549 cells; correspondingly, depletion significantly reduced the number and the size of foci in H460 cells **(B)**. Wound healing assay results showed that Lasp1 transfection in A549 cells resulted in longer distances traveled, while reducing Lasp1 expression in H460 cells by siRNA transfection resulted in shorter **(C)**. Representative images of cells on the bottom of Transwell membranes show the changes in invasive cell numbers **(D)**, ×400, scale bar= 50 μm) (*, *P*<0.05; **, *P*<0.01, ***, *P*<0.001).

Next, we investigated the effect of Lasp1 on the proliferation and invasion abilities after transfecting Lasp1 plasmid in A549 cells or transfecting Lasp1 siRNA in H460 cells. MTT assay and colony formation assay results revealed that overexpressing Lasp1 promoted proliferation and colony formation abilities of A549 cells, and depleting Lasp1 inhibited proliferation and colony formation abilities of H460 cells (Figure 2A and 2B). Results of wound healing test and transwell assay suggested that tumor migration and invasion were enhanced after transfecting Lasp1 plasmid in A549 cells, but were depressed after transfecting Lasp1 siRNA in H460 cells (Figure 2C and 2D).

### Lasp1 increased CyclinA2, CyclinB1, and Snail, and decreased E-cadherin expression

**Figure 3:**
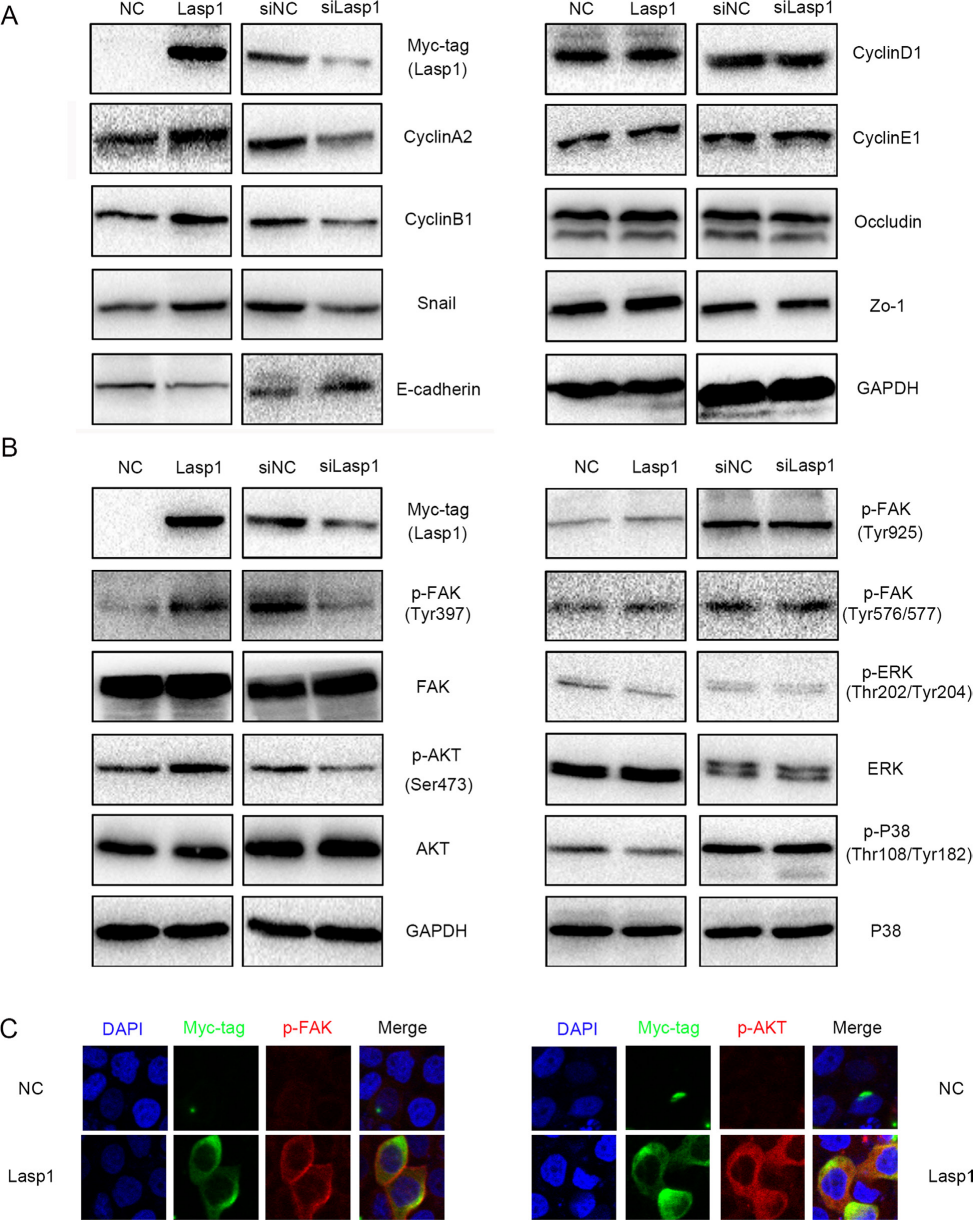
Overexpressing Lasp1 increased the expression of CyclinA2, CyclinB1, Snail and decreased the expression of E-cadherin CyclinA2, CyclinB1, Snail expressions were upregulated, whereas E-cadherin expression was downregulated after overexpressing Lasp1 in A549 cells; correspondingly, CyclinA2, CyclinB1, Snail expressions were down-regulated, E-cadherin expression was upregulated by depleting Lasp1 in H460. The other proteins showed no visible changes **(A)**. The levels of p-AKT (Ser473) and p-FAK (Tyr397) were increased after overexpressing Lasp1 in A549 cells and were decreased after depleting Lasp1 in H460 cells; the other key signaling pathway presented no significant changes **(B)**. The immunofluorescence assay results showed that the expression of p-FAK and p-AKT were increased after transfected with Lasp1 plasmid. Magnification, 600× **(C)**.

Then, we detected the expression of cell cycle and EMT (epithelial-mesenchymal transition) related proteins after transfecting Lasp1 plasmid in A549 cells or transfecting Lasp1 siRNA in H460 cells. Overexpressing Lasp1 increased CyclinA2, CyclinB1, and Snail expression, but decreased the expression of E-cadherin (Figure 3A). Correspondingly, depleting Lasp1 decreased CyclinA2, CyclinB1, and Snail expression, but increased E-cadherin expression (Figure 3A). The other proteins, (CyclinD1, CyclinE1, Occludin and Zo-1) showed no visible changes. We next screened key signaling pathway regulators involved in modulating cell cycle and EMT. The phosphorylation of FAK in Tyr397 and phosphorylation of AKT in Ser473 was significantly elevated after transfecting Lasp1 plasmid in A549 cells and was significantly depressed after transfecting Lasp1 siRNA in H460 cells (Figure 3B). The other key signaling proteins (FAK, AKT, p-FAK-Tyr925, p-FAK-Tyr576/577, p-ERK-Thr202/Tyr204, ERK, p-p38-Thr108/Tyr182 and p38) showed no obvious alteration (Figure 3B). Immunofluorescence results showed that the levels of both p-FAK and p-AKT were enhanced after transfected with a Lasp1 plasmid in A549 cells. Either p-FAK or p-AKT was co-localized with Lasp1 in the cytoplasm (Figure 3C).

### Lasp1 enhanced cyclin and EMT proteins via activating FAK-AKT pathway

**Figure 4:**
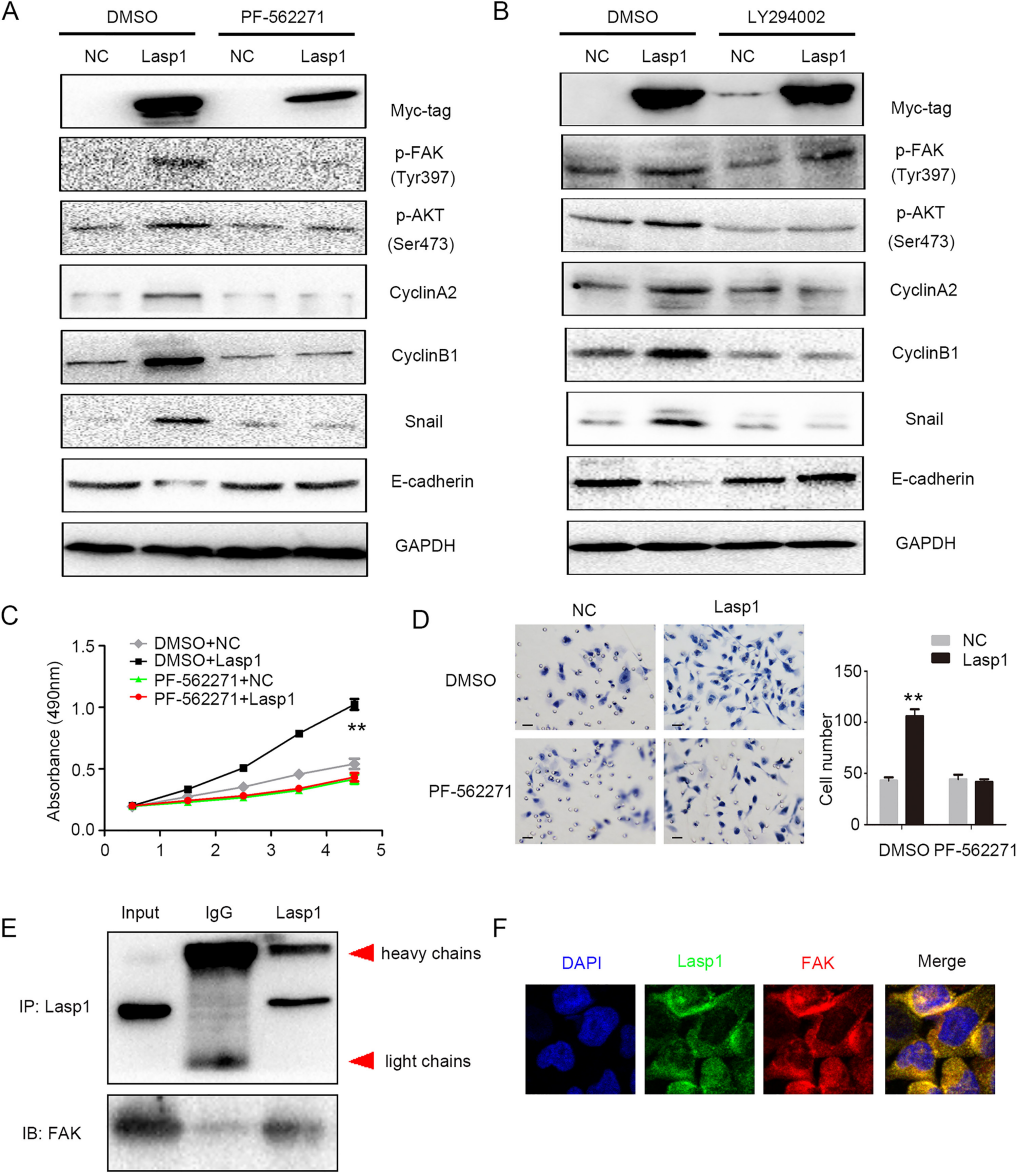
Lasp1 enhanced cyclin and EMT proteins via activating FAK-AKT pathway Treatment of FAK inhibitor (PF-562271) markedly prevented the phosphorylation of FAK and AKT and subsequently counteracted increasing expression of CyclinA2, CyclinB1, Snail and decreasing expression of E-cadherin caused by Lasp1 overexpression **(A)**. LY294002, an inhibitor of AKT, attenuated the levels of p-AKT but not p-FAK, and thereby reduced the upregulating expression of CyclinA2, CyclinB1, Snail and reversed the inhibition of E-cadherin expression induced by Lasp1 cDNA transfection **(B)**. Tumor proliferation **(C)** and invasion **(D)**; Magnification, 400×; scale bar=50μm) caused by Lasp1 overexpression were also reversed by FAK inhibitor incorporation. Immunoprecipitation assay showed that Lasp1 directly interacted with FAK **(E)**. Immunofluorescence assay showed that Lasp1 co-localized with FAK in the cytoplasm of H460 cells. Magnification; 600× **(F)**.

PF-562271, a specific inhibitor of FAK, was added into the medium after overexpressing Lasp1 in A549 (lung adenocarcinoma) and LK2 (squamous cell lung carcinoma) cells. Treatment of FAK inhibitor markedly prevented the phosphorylation of FAK and AKT and subsequently counteracts increasing expression of CyclinA2, CyclinB1, Snail and decreasing expression of E-cadherin which mediated by Lasp1 overexpression (Figure 4A, Supplementary Figure 1). Interestingly, AKT inhibitor only attenuated the levels of p-AKT and thereby counteracted the increasing expression of CyclinA2, CyclinB1, Snail and restored the decreasing expression of E-cadherin but not the levels of p-FAK which induced by Lasp1 overexpression (Figure 4B). Tumor proliferation and invasion mediated by Lasp1 overexpression was also reversed by FAK inhibitor incorporation (Figure 4C and 4D). In H460 cells, co-immunoprecipitation and immunofluorescence results suggested that Lasp1 directly interacted with FAK and co-localized in the cytoplasm (Figure 4E and 4F).

## DISCUSSION

Our results found that Lasp1 overexpressed in the cytoplasm of NSCLC tissues and associated with larger tumor size, advanced TNM staging and positive lymph node metastasis, suggesting that Lasp1 overexpression may serve as a prognostic marker of NSCLC patients. Lasp1 directly interacted with FAK and facilitated CyclinA2, CyclinB1 and Snail expression, but inhibited E-cadherin expression via phosphorylating FAK-AKT signaling pathway and thereby promoted tumor proliferation and invasion of NSCLC cells.

Our data was consistent with the previous studies that Lasp1 was overexpressed in different types of malignant tumors and significantly correlated with proliferation and invasion [4]. Herein, we found that Lasp1 was overexpressed in the cytoplasm of NSCLC tissues. Our results were similar to previous studies in NSCLC samples [15, 23]. However, nuclear accumulation of Lasp1 is observed in diverse cancer entities [24–28]. As Lasp1 nuclear translocation required binding to its shuttle partner, the cytoplasmic distribution of Lasp1 in NSCLC may attribute to tissue-specific expression of its shuttle partner, which needed to be further elucidated [29]. Overexpression of Lasp1 has been shown to correlate with multiple clinicopathological features in various types of human cancer [11, 15, 23, 27, 30–33]. The results of the present study were consistent with the previous studies that overexpression of Lasp1 was associated with larger tumor size, advanced TNM staging and positive lymph node metastasis. Similar results were also found in squamous cell lung carcinomas but not in lung adenocarcinomas. In previous studies, overexpression of Lasp1 was identified as a prognostic factor of patients’ adverse survival in a number of cancer entities, including breast cancer, colorectal cancer, gastric cancer, hepatocellular cancer, lung cancer, etc [11, 15, 23, 27, 30–33]. Our results were consistent with previous studies that Lasp1 overexpression predicted poor overall survival in NSCLC patients. Although Zheng et al. had demonstrated Lasp1 as an independent prognosis predictor in NSCLC [15], our data shed new light on the prognostic value of Lasp1 that it may be histological-type-specific, especially in squamous cell lung carcinoma. Certainly, there were several limitations of our survival analysis. Firstly, the sample size of the subgroup of patients with squamous cell lung carcinoma was too small to get a confirmative result. Secondly, due to the limit of the information of treatment after surgery, our study cannot perform an analysis of progression-free survival. Therefore we would like to encourage other academic groups to validate our data on their own tumor collections.

Lasp1 facilitated tumor proliferation and invasion in multiple cancer entities [4]. Previous studies showed that Lasp1 promoted EMT via inducing phosphorylation of MAPK, PI3K/AKT and Smad signaling in colorectal carcinoma [21, 22]. In NSCLC, Zheng et al. described that Lasp1 enhanced tumor proliferation and invasion [15]. However, the detail molecular mechanism is unclear in NSCLC. In our study, we found that Lasp1 may promote tumor proliferation and invasion through inducing phosphorylation of FAK (Tyr397) and AKT (Ser473) and thereby upregulate the expression CyclinA2, CyclinB1 and Snail, and downregulating the expression of E-cadherin. Similar to the previous studies, Lasp1 functioned as a key mediator of EMT via inducing phosphorylation of its downstream signaling pathway such as AKT. However, our results suggest that Lasp1 may regulate AKT activation through direct binding and facilitate the phosphorylation of FAK. As it is known to us all, FAK was served as an upstream factor of AKT signaling [33, 34], our finding may provide a new sight to Lasp1 function. Since the activation of FAK signaling is shown to be regulated by c-Met signaling [35–37], we also evaluated the phosphorylation of c-Met and Gab1 after either overexpressing or depleting Lasp1. No significant changes were observed (Supplementary Figure 2).

In conclusion, the present study indicated that overexpression of Lasp1 correlated with larger tumor size, advanced TNM stage, positive regional lymph node metastasis and predicted poor prognosis of NSCLC patients. Lasp1 may be the upstream regulator of FAK/AKT signaling pathway and facilitates proliferation and invasion of NSCLC through enhancing the phosphorylation of FAK (Tyr397) and AKT (Ser 473).

## MATERIALS AND METHODS

### Patients and specimens

This study was subject to approval by the local institutional review board of the China Medical University. Tissue samples were obtained from 109 patients (68 males and 41 females) who underwent complete surgical excision at the First Affiliated Hospital of China Medical University between 2010 and 2012 with a diagnosis of lung squamous cell carcinoma or lung adenocarcinoma, 38 of 109 cases had corresponding non-cancerous lung tissues. No neoadjuvant radiotherapy or chemotherapy was done before surgery. Of the 109 patients, 33 (30.3%) were treated with platinum-based adjuvant chemotherapy, 8 (7.3%) underwent platinum-based adjuvant chemoradiotherapy, and the other 68 patients were treated outside, we did not have information about treatment. The survival of each patient was defined as the time from the day of surgery to the end of follow-up or the day of death. Histological diagnosis and grading were evaluated according to the 2015 World Health Organization (WHO) classification of tumors of the lung [38]. All 109 specimens were for histological subtype, differentiation, and tumor stage. Tumor staging was performed according to the seventh edition of the International Union against Cancer (UICC) TNM Staging System for Lung Cancer [39]. The median age of 109 patients was 60 years old (range from 29 years old to 79 years old). Of the 109 patients, 49 patients were equal to or older than 60 years old, 60 patients were younger than 60 years old. The samples included 47 squamous cell lung carcinomas and 62 lung adenocarcinomas, respectively. A total of 38 tumors were well differentiated, while 71 were classified as moderately or poorly differentiated. Lymph node metastases were present in 48 of the 109 cases. Our cohort included 83 stages I–II cases and 26 stage III cases. Among 62 adenocarcinoma cases, 24 cases had got KRAS mutation detection, only 1 cases harbored KRAS mutant, and 47 had been performed EGFR mutation detection, 21 cases harbored EGFR-mutated. Among these cases with EGFR mutation were positive (12 cases in exon 19; 9 cases in exon 21). No ALK mutation was found in all the 109 cases.

### Immunohistochemistry

Samples were fixed in 10% neutral formalin, embedded in paraffin, and sliced into 4-μm thick sections. Immunostaining was performed by the streptavidin-peroxidase method. The sections were incubated with a polyclonal Rabbit anti-Lasp1 antibody (1:100; ab191022, Abcam, Cambridge, UK) at 4°C overnight, followed by the biotinylated goat anti- Rabbit IgG secondary antibody. After washing, the sections were incubated with horseradish peroxidase-conjugated streptavidin–biotin (Ultrasensitive; MaiXin, Fuzhou, China) and developed using 3, 3-diaminobenzidine tetra-hydrochloride (MaiXin). Finally, samples were lightly counterstained with hematoxylin, dehydrated in alcohol, and mounted. Two investigators blinded to the clinical data semi-quantitatively scored the slides by evaluating the staining intensity and percentage of stained cells in representative areas. The staining intensity was scored as 0 (no signal), 1 (weak), 2 (moderate), or 3 (high). The percentage of cells stained was scored as 0 (no signal), 1 (1–25%), 2 (26–50%), 3 (51–75%), or 4 (76–100%). A final score of 0–12 was obtained by multiplying the intensity and percentage scores. Tumors were seen as a positive Lasp1 expression with a score ≥4. Tumor samples with scores between 1 and 3 were categorized as showing weak expression, whereas those with scores of 0 were considered to have no expression; both weak expression and no expression were defined as a negative Lasp1 expression.

### Cell culture

The HBE cell line was obtained from the American Type Culture Collection (ATCC; Manassas, VA, USA). The A549, H460, H292, H661, H1299, and LH7 cell lines were obtained from the Shanghai Cell Bank (Shanghai, China). The human lung ADC Anip973 (973) and AGZY83a (83A) cell lines were purchased from Shanghai Bioleaf Biotech Co., Ltd (http://www.bioleaf.com) and stored in the Department of Pathology, Harbin Medical University. The LK2 cell line was a gift from Dr. Hiroshi Kijima (Department of Pathology and Bioscience, Hirosaki University Graduate School of Medicine, Japan). The information of the histological types in all cell lines was summarized in Supplementary Table 3. All cells were cultured in RPMI 1640 (Invitrogen, Carlsbad, CA, USA) supplemented with 10% fetal bovine serum (Invitrogen), 100 IU/ml penicillin (Sigma), and 100 μg/ml streptomycin (Sigma), and passaged every other day using 0.25% trypsin (Invitrogen).

### Western blot

Total protein was extracted using a lysis buffer (Pierce, Rockford, IL, USA) and quantified with the Bradford method [40]. Fifty μg of the total protein samples were separated by 10% SDS-PAGE and transferred onto polyvinylidene fluoride membranes (PVDF; Millipore, Billerica, MA, USA). Immunoprecipitation assays were performed as described previously [41]. Membranes were incubated overnight at 4°C with the following primary antibodies: Lasp1 (1:100, Abcam, Cambridge, UK); GAPDH (1:5000, Sigma, St. Louis, MO, USA); Myc-tag, CyclinD1, CyclinB1, CyclinA2, CyclinE1, Snail, p-ERK, ERK, p-AKT-Ser473, AKT, p-p38, p38, p-FAK-Tyr397, p-FAK-Tyr925, p-FAK-Tyr576/577, FAK, p-Met-Tyr1234/1235, p-Met-Tyr1349, p-Met-Tyr1003, Met, p-Gab1-Tyr307, Gab1 (1:1000; Cell Signaling Technology, Danvers, MA, USA); E-cadherin(1:1000; BD Transduction Laboratories, Lexington, KY, USA); Zo-1 and Occludin(1:500; Proteintech, Chicago, IL, USA). PF-562271 was purchased from Selleck Chemicals (Houston, TX, USA), LY294002 was purchased from Cell Signaling Technology. Membranes were washed and subsequently incubated with peroxidase-conjugated anti-mouse or anti-rabbit IgG (Santa Cruz Biotechnology) at 37 °C for 2 hours. Bound proteins were visualized using electrochemiluminescence (Pierce, Rockford, IL, USA) and detected with a bio-imaging system (DNR Bio-Imaging Systems, Jerusalem, Israel).

### Plasmid transfection and small interfering RNA treatment

Plasmids pCMV6-ddk-myc and pCMV6-ddk-myc-Lasp1 were purchased from Origene (RC219975, Rockville, MD, USA). Lasp1-siRNA (sc-105607) and NC-siRNA (sc-37007) were purchased from Santa Cruz Biotechnology. Transfection was carried out using the Lipofectamine 3000 reagent (Invitrogen) according to the manufacturer’s instructions.

### Matrigel invasion

Cell invasion assay was performed using a 24-well transwell chamber with 8 μm pores (Costar, Cambridge, MA, USA). The inserts were coated with 20μl Matrigel (1:3 dilution; BD Bioscience, San Jose, CA, USA). Forty-eight hours after transfection, cells were trypsinized, and 3×10^5^ cells in 100 μl of serum-free medium were transferred to the upper Matrigel chamber for 18 hours. Media supplemented with 10% FBS were added to the lower chamber as a chemoattractant. After incubation, cells that passed through the filter were fixed with 4% paraformaldehyde and stained with hematoxylin. The invasive cells were microscopically counted in 10 randomly selected high-power fields.

### Wound healing assay

In cultures with cell density below 90%, 48 hours after transfection, wounds were created in confluent areas using a 200-μl pipette tip. Wound healing within the scrape line was observed at different time points, and representative scrape lines for each cell line were photographed. Duplicate wells were examined for each condition, and each experiment was repeated 3 times. The optical wound distances were measured using Image J software (National Institute of Health, Bethesda, MD, USA).

### MTT

Cells were plated in 96-well plates in medium containing 10% FBS at about 3000 cells per well 24 hours after transfection. For quantitation of cell viability, cultures were stained after 4 days by using the MTT assay. Briefly, 20 μl of 5 mg/ml MTT (Thiazolyl blue) solution was added to each well and incubated for 4 hours at 37°C, then the media was removed from each well, and the resultant MTT formazan was solubilized in 150 μl of DMSO. The results were quantitated spectrophotometrically by using a test wavelength of 490 nm.

### Colony formation assay

The A549 and H460 cells were transfected with pCMV6 or pCMV6-Lasp1 plasmids, negative control or Lasp1-siRNA for 48 hours. Thereafter, cells were planted into three 6-cm cell culture dishes (1000 per dish for A549 and H460 cell lines) and incubated for 12 days. Plates were washed with PBS and stained with Giemsa. The number of colonies with more than 50 cells was counted. The colonies were manually counted by microscope.

### Immunofluorescence staining

Cells were fixed with 4% paraformaldehyde, blocked with 1% bovine serum albumin, and incubated overnight with Lasp1, p-FAK, p-AKT, FAK, Myc-tag antibodies (1:100) at 4°C. Cells were then incubated with tetramethylrhodamine isothiocyanate-conjugated secondary antibodies (Cell Signaling Technology) at 37°C for 2 h. Cell nuclei were counterstained with 4′, 6-diamidino-2-phenylindole (DAPI). Epifluorescence microscopy was performed using an inverted Nikon TE300 microscope (Nikon Co., Ltd., Tokyo, Japan), and confocal microscopy was performed using a Radiance 2000 laser scanning confocal microscope (Carl Zeiss, Oberkochen, Germany).

### Statistical analysis

SPSS version 22.0 for Windows (SPSS, Chicago, IL, USA) was used for all analyses. The Pearson’s Chi-square test was used to assess possible correlations between Lasp1 and clinicopathological factors. Kaplan–Meier survival analyses were carried out in 109 cases specimens and compared using the log-rank test. Mann-Whitney U test was used for the image analysis of western blot results and the invasive assay results. P<0.05 was considered to indicate statistically significant differences.

## SUPPLEMENTARY MATERIALS FIGURES AND TABLES


