# Supplementary material for: Lasp1 promotes malignant phenotype of non-small-cell lung cancer via inducing phosphorylation of FAK-AKT pathway
Source: Oncotarget. 2017 Aug 24;8(43):75102–13. doi: 10.18632/oncotarget.20527 (PMC5650404; doi:10.18632/oncotarget.20527)
Supplement: Supplementary file 1 [file oncotarget-08-75102-s001.pdf]

Lasp1 promotes malignant phenotype of non-small-cell lung cancer via inducing phosphorylation of FAK-AKT pathway

SUPPLEMENTARY MATERIALS

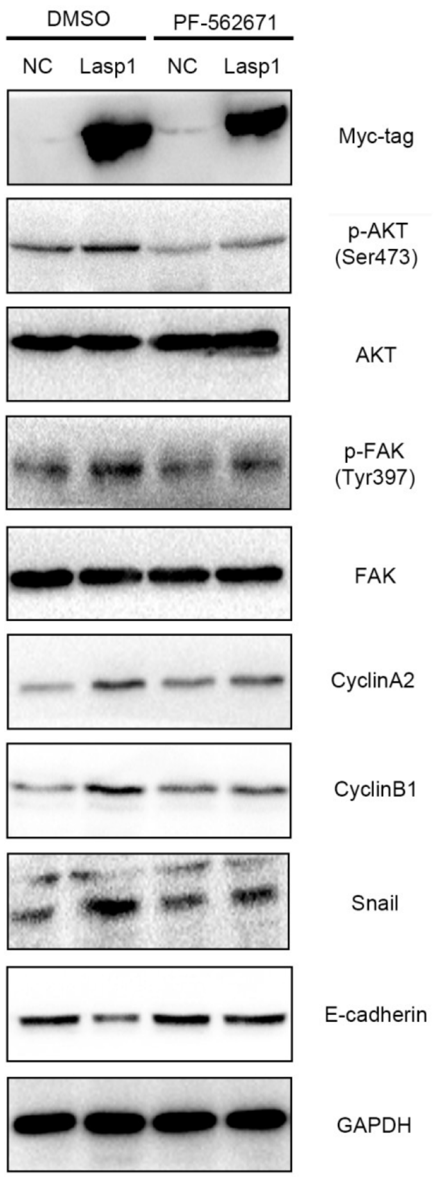

Supplementary Figure 1: Lasp1 enhanced NSCLC cell invasion and proliferation by activating FAK-AKT signaling. PF-562271, a specific inhibitor of FAK, was added into the medium after overexpressing Lasp1 in LK2 cells. Treatment of FAK inhibitor markedly prevented the phosphorylation of FAK and AKT and subsequently counteracts increasing expression of CyclinA2, CyclinB1 and Snail and decreasing expression of E-cadherin mediated by Lasp1 overexpression.

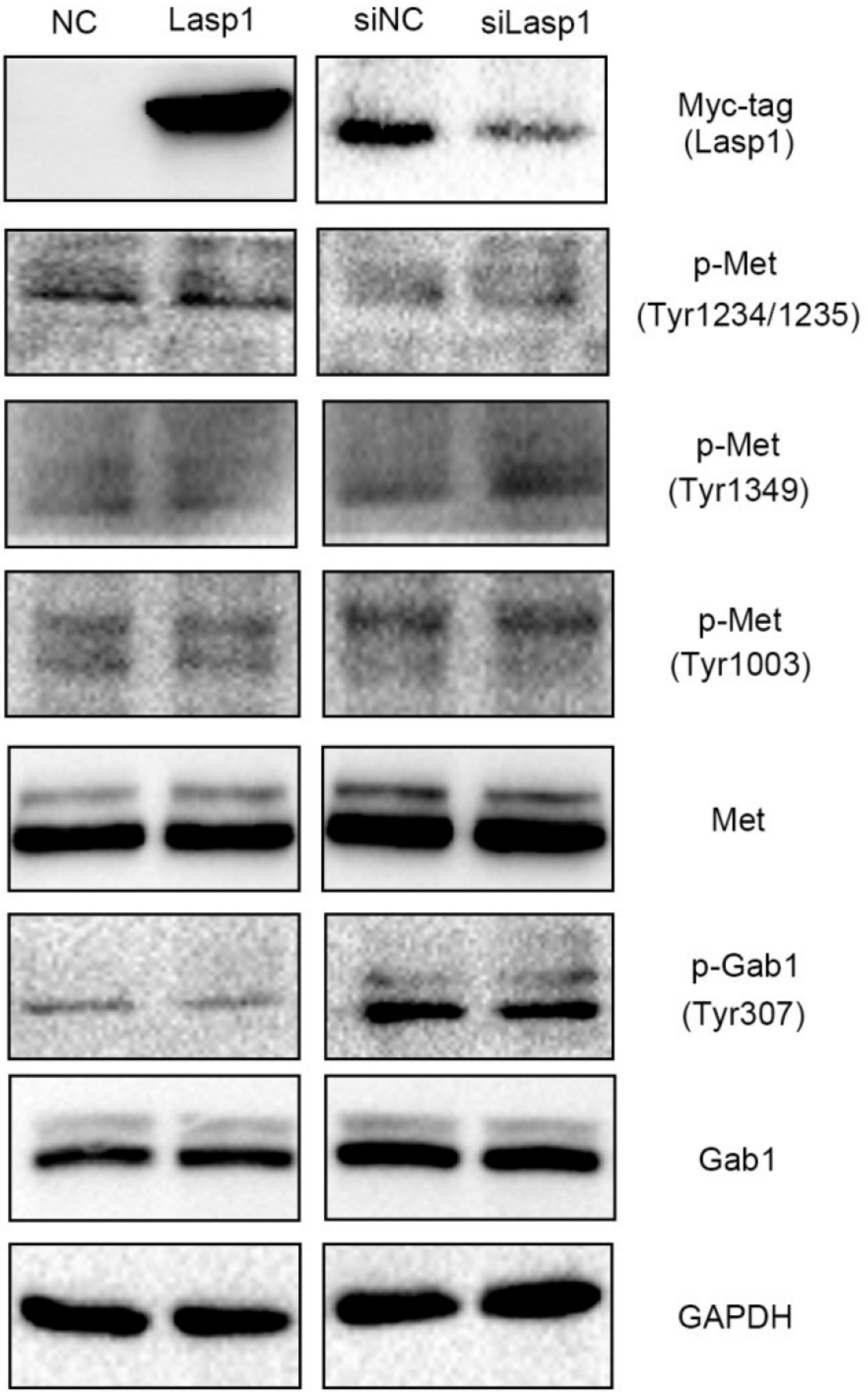

Supplementary Figure 2: The effect of Lasp1 on the Met-Gab1 signaling pathway. The protein levels of p-Met (Tyr1033, Tyr1234/1235, Tyr1349), Total Met, p-Gab1, Total Gab1 showed no significant changes when we overexpressed Lasp1 in A549 or inhibited Lasp1 with siRNA in H460 cells.

Supplementary Table 1: The association between Lasp1 expression and the clinicopathological characteristics of squamous cell lung carcinoma

		Lasp1+ (n=23)	Lasp1- (n=24)	χ2	*P*
**Age**					
	< 60	16	15	0.000	1.000
	≥ 60	16	15		
**Gender**					
	Male	20	20	0.122	0.727
	Female	3	4		
**Tumor size**					
	≤ 3cm	7	18	9.368	**0.003**
	> 3cm	16	6		
**Differetiation**					
	Well	2	5	1.365	0.416
	Moderate & Poor	21	19		
**TNM staging**					
	I-II	14	22	6.214	**0.017**
	III_A	9	2		
**Lymph node metastasis**					
	No	18	6	9.368	**0.003**
	Yes	7	16		

Supplementary Table 2: The association between Lasp1 expression and the clinicopathological characteristics of lung adenocarcinoma

		Lasp1+ (n=32)	Lasp1- (n=30)	χ2	*P*
**Age**					
	< 60	16	15	0.000	1.000
	≥ 60	16	15		
**Gender**					
	Male	18	10	3.283	0.070
	Female	14	20		
**Tumor size**					
	≤ 3cm	20	23	1.462	0.227
	> 3cm	12	7		
**Differetiation**					
	Well	14	16	0.258	0.611
	Moderate & Poor	17	15		
**TNM staging**					
	I-II	23	24	0.557	0.455
	III_A	9	6		
**Lymph node metastasis**					
	No	18	18	0.089	0.765
	Yes	14	12		
**TRU**					
	Yes	29	24	1.409	0.235
	No	3	6		
**Histological subtypes**					
	Lepidic	18	18	6.876	0.230
	Acinar	1	2		
	Papillary	7	3		
	Micropapillary	3	0		
	Solid	0	1		
	Variants	3	6		
**EGFR mutation**					
	No	13	13	0.238	0.770
	Yes	12	9		
**KRAS mutation**					
	No	14	9	1.461	0.417
	Yes	0	1		

Abbreviations: TRU, terminal respiratory unit

Supplementary Table 3: Details of histological type of NSCLC cell lines

	Type
HBE	Normal bronchial epithelial cell
LH7	Large cell carcinoma
H1299	Large cell carcinoma
H460	Large cell carcinoma
83A	Adenocarcinoma
973	Adenocarcinoma
LK2	Squamous cell carcinoma
H292	Mucoepidermoid pulmonary carcinoma
H661	Large cell carcinoma
A549	Adenocarcinoma
